# Akt Interacts with Usutu Virus Polymerase, and Its Activity Modulates Viral Replication

**DOI:** 10.3390/pathogens10020244

**Published:** 2021-02-20

**Authors:** Laura Albentosa-González, Rosario Sabariegos, Armando Arias, Pilar Clemente-Casares, Antonio Mas

**Affiliations:** 1 Centro Regional de Investigaciones Biomédicas (CRIB), Universidad de Castilla-La Mancha, C/Almansa 14, 02008 Albacete, Spain; laura.albentosa@alu.uclm.es (L.A.-G.); MRosario.Sabariegos@uclm.es (R.S.); Armando.Arias@uclm.es (A.A.); Pilar.CCasares@uclm.es (P.C.-C.); 2 Facultad de Medicina, Universidad de Castilla-La Mancha, C/Almansa 14, 02008 Albacete, Spain; 3 Unidad de Biomedicina UCLM-CSIC, Universidad de Castilla-La Mancha, C/Altagracia 50, 13071 Ciudad Real, Spain; 4 Escuela Técnica Superior de Ingenieros Agrónomos de Albacete, Universidad de Castilla-La Mancha, Campus Universitario, s/n, 02071 Albacete, Spain; 5 Facultad de Farmacia, Universidad de Castilla-La Mancha, Av. Dr. José María Sánchez Ibáñez, s/n, 02008 Albacete, Spain

**Keywords:** USUV, replicase, NS5, RNA-dependent RNA-polymerase, PI3K/Akt/mTOR pathway, inhibitors, host factors

## Abstract

Usutu virus (USUV) is a flavivirus that mainly infects wild birds through the bite of Culex mosquitoes. Recent outbreaks have been associated with an increased number of cases in humans. Despite being a growing source of public health concerns, there is yet insufficient data on the virus or host cell targets for infection control. In this work we have investigated whether the cellular kinase Akt and USUV polymerase NS5 interact and co-localize in a cell. To this aim, we performed co-immunoprecipitation (Co-IP) assays, followed by confocal microscopy analyses. We further tested whether NS5 is a phosphorylation substrate of Akt in vitro. Finally, to examine its role in viral replication, we chemically silenced Akt with three inhibitors (MK-2206, honokiol and ipatasertib). We found that both proteins are localized (confocal) and pulled down (Co-IP) together when expressed in different cell lines, supporting the fact that they are interacting partners. This possibility was further sustained by data showing that NS5 is phosphorylated by Akt. Treatment of USUV-infected cells with Akt-specific inhibitors led to decreases in virus titers (>10-fold). Our results suggest an important role for Akt in virus replication and stimulate further investigations to examine the PI3K/Akt/mTOR pathway as an antiviral target.

## 1. Introduction

Flaviviriruses are obligate intracellular parasites with a single-stranded RNA genome of positive polarity ((+)ssRNA viruses) that are grouped into a single family (*Flaviviridae*) divided into four genera (*Flavivirus*, *Hepacivirus*, *Pegivirus*, and *Pestivirus*). Of these, the genus *Flavivirus* is the one that groups the largest number of species that cause disease in humans [1,2]. Some relevant members of the genus *Flavivirus* to humans are dengue virus (DENV), Japanese encephalitis virus (JEV), yellow fever virus (YFV), West Nile virus (WNV), Zika virus (ZIKV), Usutu virus (USUV), tick-borne encephalitis virus (TBEV), and Powassan virus (POWV). Most viruses belonging to this genus are arboviruses, as they are transmitted to humans and other vertebrate hosts through the bite of arthropods, either mosquitoes or ticks [3]. Approximately 50% of these viruses are mosquito-borne, 28% are borne by ticks and the remaining 22% have no known arthropod vector to date [4]. Vaccines have been developed against some of them, providing high levels of protection in controlling infection (JEV, WNV, YFV, TBEV) [3]. Although there are some drugs approved for the treatment of flaviviral disease [5,6], most treatments available aim to mitigate the symptoms rather than inhibit virus replication and infection [7].

USUV was first identified in 1959 in South Africa, although additional studies did not begin until the first human cases were described, first in Africa in 1981 [8] and then in Europe in 2009 [9]. It belongs to the JEV serocomplex, and is genetically and serologically related to WNV, another emerging arbovirus [10]. Birds are natural vertebrate hosts for USUV, while humans are considered accidental hosts. The infection is transmitted through the bites of infected mosquitoes, primarily by the genus *Culex*. From a research point of view, USUV is an attractive pathogen as it generally requires lower biosafety containment levels than other genetically related pathogens which makes it suitable as a model to study flavivirus biology and infection [11]. In recent years, it has also become an increasingly interesting pathogen from a human health perspective, with an increase in the number of cases associated with neurological disease [12,13,14]. Some comprehensive reviews on this emerging pathogen have recently been published [15,16].

The replicative cycle of USUV appears to be very similar to that of other viruses of its genus, although it has not yet been analyzed in depth. The virus enters the cell through clathrin-mediated endocytosis, and the viral genome is subsequently released into the cytoplasm [1]. There, the viral genomic RNA is recognized by the cellular translation machinery leading to the synthesis of a single polyprotein of 3434 amino acids in size. This polypeptide is co- and post-translationally processed by host and viral proteases to yield at least eleven mature proteins termed capsid (C), premembrane (pr), membrane (M), envelope protein (E), and non-structural proteins NS1, NS2A, NS2B, NS3, NS4A, NS4B, and NS5 [15]. A dynamic and complex interplay between different viral and cellular proteins regulates each step of the viral life cycle and the re-programming of the host cell, which are critical to a complete a successful infection [17].

Flaviviral NS5 protein contains an RNA-dependent RNA polymerase domain (RdRpD) for the synthesis of new viral RNA genomes, and a methyltransferase (MTase) domain, with MTase activity, which catalyzes the capping of these new RNA molecules [18]. Flaviviral NS5 interacts with different host proteins in an infected cell. Some of these interactions are relevant to the process of viral RNA genome synthesis, while in some other cases the interaction of NS5 with other host factors can interfere with or modulate cellular functions [19]. 

Similar to other RNA viruses, flavivirus replication lacks proof-reading activities to correct replication errors [20]. Consequently, the flavivirus progeny genomes show high mutation rates which, together with their high replication rate, hinder the efficacy of antiviral activities against them. Typically, RNA viruses are very effective in evolving into variants resistant to different environmental constraints such as the presence of an antiviral drug or host immune responses [21]. An alternative approach to therapeutic drugs targeting the virus is inhibiting those cellular pathways necessary for the virus to replicate [22,23]. It is expected that such a strategy is less prone to select for resistant variants as the cellular factors are the targets for the inhibitory drugs.

Previous work by our group has shown that the PI3K/Akt/mTOR pathway is involved in the replication of hepatitis C virus (HCV), a flavivirus of the genus *Hepacivirus*. Serine-threonine kinase Akt is a protein involved in multiple cellular processes such as the regulation of metabolism, cell survival or cell proliferation among many others [24,25,26]. Akt is a key component of the PI3K/Akt/mTOR route. This route is activated by the translocation of Akt to the membrane and the phosphorylation of their Thr208 and Ser473 residues [27], and it has been described that this route is activated during infection by hepatitis C virus (HCV), WNV, DENV and JEV [28,29,30,31]. Previously, we described that the HCV polymerase interacts and co-localizes with the host cell kinase Akt. The Akt-specific inhibitor MK-2206 showed activity against HCV replication in infected cells [25]. We have recently established methods for the expression in bacteria and purification of USUV NS5 and its corresponding RdRp domain and their biochemical characterization in vitro [32]. Owing to these precedents, here we have analyzed whether USUV replication is regulated by Akt. We found that a purified USUV NS5 protein is phosphorylated in vitro by Akt, and that both proteins interact and co-localize in vivo. Furthermore, specific drugs against Akt inhibit USUV replication during infection in cell culture.

## 2. Results

### 2.1. NS5 and Its RdRp Domain Are Phosphorylated by and Interact with Cellular Akt

**Figure 1:**
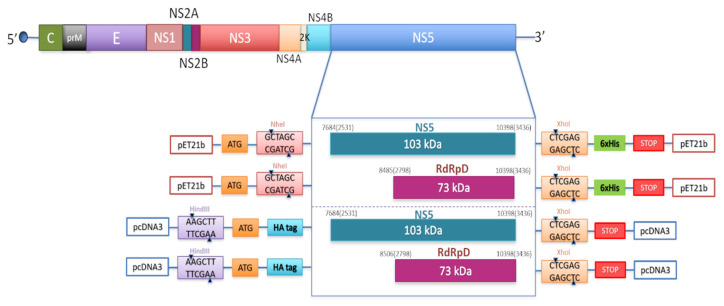
Schematic representation of the constructs and the cloning strategy used in this study.

DNA fragments spanning USUV NS5 residues encoding the full-length protein NS5 and its corresponding RdRp domain (RdRpD) were amplified by PCR and cloned into pET-21b (Novagen, Madison, WI, USA), and pcDNA3 (Figure 1).

USUV genome organization showing the polyprotein coding region (above) and the cloning strategy for the constructs used in this study (below). Constructs show two different inserts (NS5 and RdRpD) introduced into two different vectors, pET21b with the cloning sites for NheI (GCTAGC) and XhoI (CTCGAG), and a 6xHis tag, and pcDNA3 with the cloning sites for HindIII (AAGCTT) and XhoI (CTCGAG), and a HA tag in the N-terminal end. Numbers are referred to the positions of nucleotide and amino acid residues (in brackets) of NS5 and RdRp domain. The residue numbering corresponds to the USUV strain (939/01) (NCBI Reference Sequence NC_006551.1).

**Figure 2:**
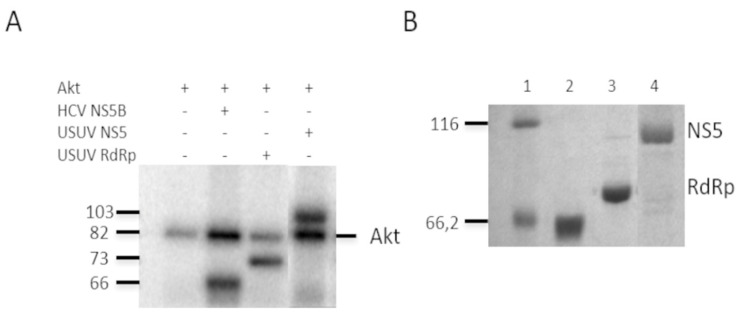
Phosphorylation of NS5 by human Akt. Panel A shows in vitro phosphorylation by Akt of the recombinant proteins HCV NS5B as a positive control, USUV RdRp domain, and USUV NS5. Reactions were resolved in one 10% SDS-PAGE gel. The bands corresponding to the autophosphorylation of Akt (line Akt) are indicated on the right, and the molecular weights on the left. Panel (**B**) shows the Coomassie stain of the recombinant proteins used in the assay described in (**A**), after being resolved in another 10% SDS-PAGE gel. HCV NS5B (lane 2), USUV RdRp domain (lane 3) and USUV NS5 (lane 4) are shown. Expected molecular weights for HCV NS5B, USUV RdRpD, and USUV NS5 are 66 kDa, 73 kDa, and 103 kDa, respectively. The molecular weight of marker proteins (lane 1) is indicated on the left.

We firstly expressed and purified the USUV proteins described above to perform the in vitro kinase assays with recombinant Akt as described in Materials and Methods. We also included the HCV polymerase NS5B as a positive control for phosphorylation (Figure 2A)^15^. The mobility of the purified proteins correlated with their predicted molecular weights, 66.2 kDa for the NS5B HCV protein (lane 2), 73 kDa for the USUV RdRpD (lane 3), and 103 kDa for the NS5 USUV (lane 4). We used these proteins (Figure 2B) as substrates for in vitro phosphorylation (Figure 2A) reactions using recombinant Akt and ^32^P-labeled ATP and showed (Figure 2B) that all of them were phosphorylated in at least one Ser or Thr position. Because Akt is self-phosphorylated we also observed a band that corresponds to the molecular weight of this protein in all lanes (Figure 2A, lane 1).

**Figure 3:**
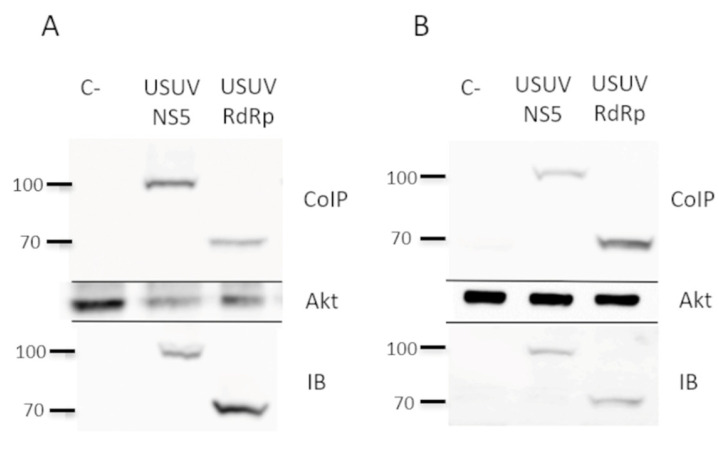
Interaction of NS5 and Akt in cell culture. Co-immunoprecipitation experiments using extracts from Huh7.5 (**A**) and Hek-293T cells (**B**), previously transfected with pcDNA3-USUV NS5 or pcDNA3-USUV RdRpD. As a negative control (C-) in both panel A and B, we have included protein lysates from non-transfected cells that were grown in parallel to the transfected cultures. Cell extracts from both transfections and negative control were immunoprecipitated with an anti-Akt antibody, followed by immunoblotting with an anti-HA antibody (upper panel). To confirm the presence of Akt, the same membranes were stripped and blotted again using an anti-Akt antibody, which can be seen on the Akt panel. An aliquot taken from each whole-cell lysate, which were later used in the co-immunoprecipitation experiments, was immunoblotted with anti-HA (IB panel).

In vitro phosphorylation of a protein by a cellular kinase implies that both proteins may be interacting at some point, although whether such an interaction is also occurring in a cellular environment remained unclear. To investigate whether the viral (USUV NS5 or RdRpD) and the cellular protein (Akt) interact inside a human cell, we expressed the viral proteins (NS5 or RdRpD) in cell culture by using the pcDNA3 constructs (Figure 1) and analyzed if they co-immunoprecipitate with Akt. To increase the significance of this analysis, we used two different cell lines in parallel, Huh7.5 (Figure 3A) and HEK293T (Figure 3B). As shown in Figure 3, these proteins were expressed in cells of human origin and could be detected by immunoblotting with anti-HA antibodies (IB in Figure 3A,B). When the cellular extracts were immunoprecipitated with anti-Akt antibodies, we confirmed the presence of USUV NS5 and its RdRp domain among the pulled-down proteins (Co-IP in Figure 3A,B). These experiments demonstrated that Akt phosphorylates both USUV NS5 and its RdRp domain, and that such an interaction is sufficiently stable to be detected by co-immunoprecipitation approaches.

**Figure 4:**
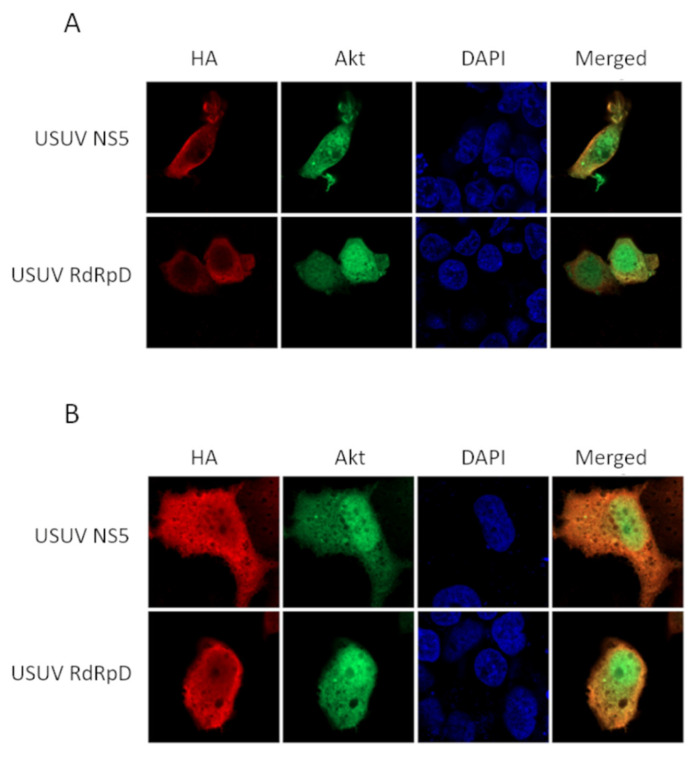
Intracellular localization of USUV NS5 or RdRpD, and Akt. (**A**) HEK-293T cells were transiently transfected adding two plasmids; one encoding Akt (pcLN-Akt-GFP) and another with the USUV protein (pcDNA3-USUV_NS5 or pcDNA3-USUV_RdRpD). The immunocytochemistry experiments were performed using antibodies against HA, showed in red, detecting USUV NS5 and USUV RdRpD. DAPI (in blue) was used to detect cell nuclei. Akt is expressed as a GFP-fusion protein, and hence a green signal associated with this protein is detected. On the right panel, we can see the merge of red and green channel images. Merged images show an overlay with co-localization in orange. (**B**) Same experiment with Huh-7.5 cells.

Then, we investigated whether NS5 and its RdRpD co-localize with Akt in human cells, and if so, in which cellular compartment this interaction is taking place. For this purpose, we transfected Huh7.5 and HEK293T cells with the constructs expressing USUV proteins and analyzed by confocal microscopy the location of NS5 or the RdRpD, and the Akt cell kinase, as described in Materials and Methods. These results clearly show that, independently of the cell line used, both viral and cellular proteins merge in the cytoplasm of the transfected cell (Figure 4). Although the Akt signal is abundantly detected in the nucleus, and while the flaviviral NS5 protein and its RdRp domain can also be found in the nuclei of transfected cells, lower nuclear co-localization of both proteins is observed. This is the case for both HEK293T cells, in which the NS5 and RdRpD signals are only detected in the cytoplasmic region, and Huh7.5 cells, in which NS5 and RdRpD signals are partly observed in the nucleus (Figure 4).

### 2.2. Effect of Akt Inhibition on USUV Replication

Finally, we investigated whether inhibition of Akt pathways, by drugs with different specificity and mechanism of action, had any effect on USUV infectivity kinetics in cell culture. We used three different drugs. Two of them are specific inhibitors for Akt: ipatasertib (a competitive molecule) and MK-2206 (a non-competitive compound). We also tested honokiol, a non-specific inhibitor of Akt, which also inhibits other cell signaling pathways. We first determined the toxicity of each drug in Vero cells, as they were later used in the infection assays, by testing increasing concentrations of these compounds.

**Figure 5:**
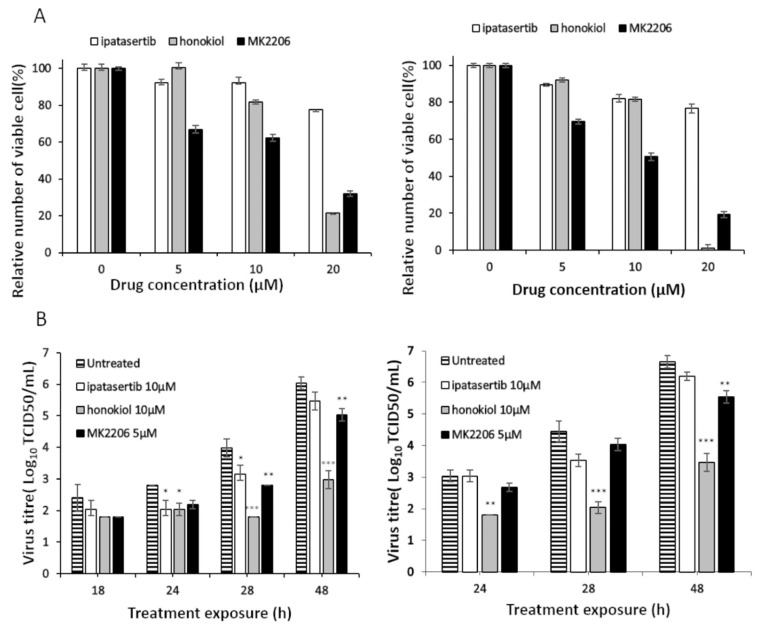
Effect of Akt inhibitors on the replication of USUV in cell culture. (**A**) Viability of Vero cells in the presence of ipatasertib, honokiol, and MK-2206. The relative number of viable cells in plates treated with Akt inhibitors for 24 h (left) or 48 h (right) is shown (100% of viable cells correspond to cells grown in the absence of inhibitors). (**B**) Virus titer (Log_10_TCID50/ml) in the absence and in the presence of Akt inhibitors. Inhibitors were added 5 h before infection (left) or at the time of infection (right) and virus titer calculated as described in Materials and Methods at different treatment times. Virus titers for untreated cells, and for cells treated with MK-2206, honokiol, and ipatasertb are shown. Values are the averages from three determinations. Standard deviation values are smaller than the graph symbols and are, therefore, not visible. Statistical analysis of USUV sensitivity to each drug compared to infections in mock-treated cells was determined by two-way ANOVA test followed by Dunnett’s correction for multiple comparisons, *p* < 0.05, *; *p* < 0.01, **; *p* < 0.001,***.

Vero cells (originating from African green monkey cells) are commonly used in the propagation of most flaviviruses as they generally allow efficient replication accompanied with elevated viral yields. The sequence of the homologous protein in Old World monkeys is nearly identical to human Akt (>99%), and hence should be targetable by the same inhibitors. From these experiments we decided to use 5 µM MK-2206 and 10 µM ipatasertib and honokiol in cell culture infection assays, since these concentrations result in a relative number of viable cells above 65% both for 24- (Figure 5A left panel) and 48-hour treatments (Figure 5A right panel).

Then, we infected Vero cells with USUV in the presence and absence of these inhibitors following two different strategies: either pretreating the cells for 5 h before infection or beginning the treatment at the time of infection. This approach may reveal any effect of Akt inhibition on the early events of the virus in the cell. As shown in Figure 5B, honokiol is the most effective drug against USUV as it resulted in further decreases in virus titers, leading to more than a 3-Log_10_ reduction using both treatment strategies 48 hours after treatment (Figure 5B). The other two inhibitors showed milder effects on USUV replication. MK-2206 resulted in modest but significant decreases of up to 1 Log_10_ in virus titers in both pretreated and non-pretreated cells, and these differences can be seen within 48 hours in both conditions and at 24 h in pre-treatment conditions.

Although treatment with ipatasertib shows a trend of decreasing viral titers compared to controls, showing a 1 Log_10_ decrease in virus titer only in cells pre-treated with the drug, there was no significant difference at any time of sample collection (Figure 5B).

## 3. Discussion

Here we demonstrate a connection between the activity of the Akt kinase and USUV polymerase which is important for viral replication in the infected cell. We found that the USUV NS5 protein interacts with and is phosphorylated by the cellular kinase Akt. Flaviviral polymerases could be considered as multifunctional proteins because they replicate the viral genome and also interact with host factors to modulate cellular physiology. This role has been documented for several members of the *Hepacivirus* and *Flavivirus* genera. Specifically, the polymerase of the hepaciviruses (i.e., HCV) establishes critical interactions with cellular factors that are involved in cell cycle control such as the retinoblastoma protein [33], VAP-B and VAP-C [33,34], cyclin A2 [35], c-Src [36], TRiC/CCT [37], nucleolin [38], estrogen receptor [39], and Hsp72 [40], among others. Some of these interactions have been described in depth, such as NS5B with cyclophilin B, nucleolin [38], estrogen receptor [39], and Hsp72 [40], among others. Some of these interactions, such as NS5B with cyclophilin B [41], have allowed for establishing antiviral strategies for the control of HCV infection [42].

In a similar way, the interaction of NS5 proteins from members of the *Flavivirus* genus, such as DENV and ZIKV, with cellular factors play a regulatory function both in the replication of the viral genome and in the control of the cell physiology. Thus, the interaction of NS5 with cyclophilins and the corresponding inhibition by cyclosporins [43], Hsp70 [44], and with a great number of proteins related to the regulation of the immune system has been described [45]. In fact, most flavivirus NS5 interactions described so far are with proteins related to the interferon response [46], such as Hsp90 and JAK/STAT, among others [47,48,49,50]. It seems that the nuclear location of NS5 is not relevant to this type of modulation of the immune response [51]. Some of these routes have common control points with the PI3K/Akt/mTOR route [52,53]. The Akt protein is part of the PI3K/Akt/mTOR pathway, with implications in immunomodulation, metabolism, and apoptosis [54,55,56,57,58,59,60,61]. The participation of Akt in the activation of dynamin and the subsequent participation in the formation of endocytosis vesicles, which is an important mechanism for membrane protein downregulation, synaptic communication, and virus entry, has also been described [62,63,64,65,66,67].

We predict that the interaction of USUV NS5 with Akt could be affecting the activity of Akt, NS5, or both proteins. Phosphorylation of NS5 could be altering RNA-polymerase and/or MTase activities but also could be acting as a molecular switch to modulate the binding of other cellular or viral partners. It is also expected that NS5 phosphorylation in Ser and/or Thr residues could be affecting or even abrogating RdRp activity as it has been previously described for norovirus and HCV polymerases [68,69]. All these constitute exciting open possibilities that are currently being investigated in our group. Furthermore, a phosphorylated NS5 could also have a different conformation, allowing its interaction with other proteins to regulate cellular processes relevant to viral replication. We have previously documented that USUV NS5 is imported to the nucleus [32] and this transport could also be regulated by Akt phosphorylation. The phosphorylation of other viral polymerases by this and other cellular kinases implies, in most cases, the loss of RdRp activity [68,69] and the modulation of viral replication [25,70]. These polymerases (HCV, norovirus) do not have the ability to translocate to the cell nucleus, while other flavivirus (e.g., USUV) do [32], adding an additional level of complexity to flavivirus-cell interactions and modulation of infection.

The specific Akt inhibitors used in this study have different mechanisms of action. Ipatasertib is a competitive inhibitor that binds to the ATP binding site [71]. MK-2206 is an allosteric inhibitor, and hence does not bind to the ATP site [72]. Even though the mechanism of action of MK-2206 is not completely understood, existing data suggest that its binding induces a closed conformation that occludes interacting sites for activating kinases (PDK1, mTORC2) and Akt substrates, which also disrupts membrane localization of Akt [73]. The differences we found in the inhibition profiles could be due to a different mechanism of action of these two inhibitors. Regardless of the specific drug (MK-2206 or ipatasertib) used in the assay, it appears that the effect was modestly larger when the cells were pre-treated with the drug. Ipatasertib and MK-2206 have been broadly investigated as anticancer drugs, including in several clinical trials, which encourage their repurposing or redevelopment as potential antiviral molecules. In the light of our results, honokiol elicits greater antiviral activity than MK-2206 and ipatasertib. Nonetheless, it remains unclear whether this effect is linked to Akt inhibition alone, or it can be due to additional inhibitory activities associated. Honokiol is a non-specific inhibitor of the PI3K/Akt/mTOR route which also targets different cellular signaling pathways. Hence, its possible therapeutic use in the treatment of infection might be compromised, owing to potential undesirable non-targeted side effects associated with it [74].

As can be seen from our results, Akt drug inhibitors led to reduced numbers of viable cells (1.2- to 1.5-fold reduction) with respect to untreated cultures. It could be argued that the antiviral effect associated could be partially due to reduced viability in these experiments. We deem, however, that such a relatively modest decrease in the number of cells cannot be accounting for larger drops (10- to 1000-fold) in virus titers. The viability assay used in this study (Cell-Titer Blue) only detects fully metabolically active cells that in principle should be as susceptible to infection as in untreated replicas. Since all these compounds are anticancer drugs, which strongly inhibit cell division [75], it is conceivable that this reduction in the number of cells is a reflection of lower division rates rather than major toxicity issues that could be affecting viral replication. 

In summary, here we have documented that the USUV protein NS5 interacts with and is phosphorylated by the cellular kinase Akt. This interaction is important for the replicative cycle of the virus, since specific Akt drugs could reduce USUV replication in cultured cells. In addition, the virus titer after treatment was different depending on the type of inhibitor, which will allow future studies to explore the underlying mechanism. This work stimulates further study on Akt and related cellular factors as possible therapeutic targets in the treatment of flavivirus infections. Further experiments with these and other Akt inhibitor drugs may lead to new therapeutic possibilities in the treatment of infection. In particular, we anticipate that new Akt drugs with improved toxicity profiles, or combinations of small molecule compounds targeting different Akt motifs, may lead to a better disruption of the Akt-NS5 interaction, and hence improve the antiviral efficacy. Alternative specific Akt inhibitors to those used in this study include uprosertib and perifosine, both of which have been tested in several cancer clinical trials. Drugs targeting different components of the PI3K/Akt/mTOR axis can also be instrumental in unraveling the importance of this route in USUV replication, and its potential value as a therapeutic target.

## 4. Materials and Methods

### 4.1. DNA Amplification by PCR, Cloning, and Purification

The coding sequences corresponding to the NS5 and RdRp domain (RdRpD) of USUV, were amplified by PCR, using PfuTurbo DNA polymerase (Agilent Technologies, Santa Clara, CA, USA), specifically designed primers (Table 1) and a full-length USUV cDNA as a template for USUV constructions from which we extracted the coding sequences. The NS5 and RdRpD fragments were cloned into eukaryotic and bacterial expression vectors (pcDNA3 and pET21b, respectively) using the restriction enzyme sites added to the primers. The pcDNA3 constructs contain an HA tag, added by means of the forward primers. The constructs in bacterial expression vectors were designed to contain 6x-His tags at their C-termini. The presence of the expected coding and regulatory sequences, and the absence of unwanted mutations in them were confirmed for each construct by DNA sequencing. NS5 and RdRp domain proteins were overexpressed and purified in *E. coli* as previously described [32]. The identity of these proteins was confirmed by sequencing (data not shown). Both purified NS5 and RdRpD proteins were further analyzed by immuno-blotting, using anti-6xHis antibodies.

### 4.2. In Vitro Phosphorylation by Akt

Each reaction contained 1 µL of Akt (0.22 mg/mL, Biaffin GmbH & Co KG, Kassel, Germany), DTT 0.1 M, 1 µL of γP^32^-ATP (3000 Ci/mmol), kinase buffer 1× (20 mM HEPES [pH 7.4], 10 mM MgCl_2_, 10 mM MnCl_2_), and approximately 1 µg of the purified proteins in a final volume of 30 µL. The reactions were incubated for 30 min at 30 °C, and then the reaction was stopped by adding 5 µL of protein loading buffer (300 mM Tris pH 6.8, 50% *v/v* glycerol, 10% (*v/v*) SDS, 0.05% *v/v* Bromophenol Blue). Proteins were then denaturized by heating during 5 minutes at 95 °C. Aliquots of 15 µL each were resolved in a 10% SDS-PAGE gel. The gel was exposed on a phosphorimager screen and was subsequently read in a Typhoon 9600 instrument.

### 4.3. Western-Blot and Co-Immunoprecipitation

For Western blot (WB) and co-immunoprecipitation assays we used human cell lines Huh7.5 and Hek-293T. Cells were seeded on 100 mm-cell culture plates; when the cell monolayer reached ~70% confluence, it was transfected with the appropriate plasmid construct, using Lipofectamine 3000 (Thermo Fisher Scientific, Madrid, Spain) and following the manufacturer’s instructions. On the next day after transfection, the cell culture medium was removed and complete medium added. At 48-h post-transfection, the cells were washed with PBS (Dulbecco’s Phosphate Buffered Saline-DPBS) and lysis buffer (JNK 1X buffer (25 mM HEPES [pH 7.5], 0.3 M NaCl, 1.5 mM MgCl_2_, 0.2 mM EDTA, 1% Triton X-100, 0.1% SDS, 0.5% deoxycholic acid); PMSF 0.1 M; protease inhibitor cocktail from Sigma-Aldrich; dithiothreitol 1 M) was added. The cells were collected and centrifuged (3000 rpm for 30 min at 4 °C). The protein concentration in the extract was determined by using the Pierce BCA Protein Assay kit and measuring the absorbance of each sample at 562 nm (Thermo Scientific, Madrid, Spain).

For WB experiments, 50 µg of each sample extract were resolved into a 10% SDS-polyacrylamide gel and then transferred to an Immobilon®-P nitrocellulose membrane (Millipore, Molsheim, France), using the Pierce Power Blot Cassette (Thermo Scientific, Madrid, Spain) according to the manufacturer’s instructions.

The membranes were blocked with 10% nonfat dry milk in TTBS (Tris-HC l50 mM pH 7.4, NaCl 150 mM, Tween 20 0.1% (*v/v*)) and then, the membranes were incubated in the presence of the primary antibody overnight at 4 °C. Membranes were washed thrice with TTBS, incubated with the corresponding secondary antibody, and washed thrice again with TTBS, before developing the membrane with the SuperSignal West Pico Plus Chemiluminiscent Substrate kit (Thermo Scientific, Madrid, Spain). The membrane was then analyzed with ImageQuant LAS 4000 system (GE Healthcare Bio-Sciences, Uppsala Sweden).

For Co-Immunoprecipitation (Co-IP) assays, a minimum amount of 100 µg of extract was incubated with 1 µg of primary a-Akt antibody (Akt (pan)(11E7) Rabbit mAb, Cell Signalling) for 2 h at 4 °C. Then, ~30 µL of Protein G (Protein G Sepharose 4 Fast Flow, GE Healthcare), previously washed in lysis buffer, were added to the mixture and incubated overnight at 4 °C. Immunocomplexes bound to Protein G were collected by centrifugation at 13,000 rpm and 4 °C for 1 minute. Supernatants were discarded and the sediment was washed in 1 mL of lysis buffer, and centrifuged again for 1 min at 13,000 rpm and 4 °C. To detect our USUV polymerase constructs in the immunoprecipitate we performed WB assays, using a-HA (Purified anti-HA.11 Epitope Tag Clone 16B12, BioLegend, San Diego, CA, USA) as primary antibody, and as a secondary antibody we used an a-Mouse IgG, HRP-linked Antibody (Cell Signaling). To detect cellular Akt in our pull downs, we stripped our membranes with Western Blot Stripping Buffer (TakaRa), and then performed WB assays, using as primary antibody a-Akt and as secondary a-Rabbit IgG Peroxidase conjugated (Pierce Goat).

### 4.4. Immunocytochemistry and Confocal Microscopy

To detect protein–protein co-localization, 2 × 10^5^ cells were seeded in 60 mm diameter plates, and transfected with the corresponding constructs using Lipofectamine 3000 (Thermo Fisher Scientific, Madrid, Spain). The immunocytochemistry method was performed as described in Albentosa-Gonzalez et al [32].

The experiments were analyzed in a ZEISS LSM 710 confocal microscope. The images were acquired with a format of 8-bit depth 1024 × 1024 using an oil immersion Plan-Apochromat 63X/1.40 Oil DIC M27 objective. Three channels were sequentially registered to capture Alexa546 (channel 1: 561 nm laser source and collecting 550–700 nm wavelength light), EGFP-T2 (channel 2: 488 nm laser source and collecting 495–550 nm wavelength light) and DAPI (channel 3: 405 nm laser source and collecting 427–494 nm wavelength light).

### 4.5. USUVand Protocols for Infection

The USUV strain used in this study was originally isolated from infected birds Austria 2001 and propagated seven times in Vero cells, as previously documented [76,77]. The full-genome sequence of this viral stock has been previously determined [78]. Procedures for the infection of African green monkey kidney epithelial cells (Vero cells) with USUV have been previously reported [76]. On the day preceding virus inoculation, we seeded 5 × 10^4^ cells per well in 24-well plates, and in the presence of 1 mL of complete media containing 5% *(v/v*) fetal bovine serum (FBS, Sigma, St. Louis, MO, USA), with 100 units/ml penicillin-streptomycin (ThermoFisher, Waltham, MA, USA) and adding 1 mM Hepes in high glucose DMEM (ThermoFisher, Madrid, Spain). Cells were incubated overnight at 37 °C and in the presence of 5% CO_2_. On the next day, cellular supernatants were removed and 250 µL of fresh media containing 1% FBS added to each plate. Then, 100 µL of USUV were applied to each monolayer at the multiplicity of infection (m.o.i.) indicated. Virus adsorption was allowed for 1 h at 37 °C with 5% CO_2_. The inoculum was then removed, and to eliminate unattached virus, the cells washed with complete DMEM. 1 mL of media containing 1% FBS was added to cells, and small aliquots of cellular supernatants collected at different time points post-infection. 

To analyze the antiviral behavior of PI3K/AKT/mTOR inhibitors, cells were supplemented with 1 mL of media containing 1% FBS and 5–10 µM ipatasertib [(2S)-2-(4-chlorophenyl)-1-{4-[(5R,7R)-7-hydroxy-5-methyl-5H,6H,7H-cyclopenta[d]pyrimidin-4-yl]piperazin-1-yl]-3-[(propan-2-yl)amino]propan-1-one], MK-2206[8-[4-(1-Aminocyclobutyl)phenyl]-9-phenyl-2H-[1,2,4]triazolo[3,4-f][1,6]naphthyridin-3-one] or honokiol [2-(4-hydroxy-3-prop-2-enyl-phenyl)-4-prop-2-enyl-phenol] after virus adsorption. All compounds were purchased from Selleckchem (Houston, TX, USA).

### 4.6. Virus Titration

Virus titers were determined by 50% tissue culture infectious dose (TCID50) assays [78]. Briefly, 1 × 10^4^ Vero cells in 60 µL of media with5% FBS were seeded on 96-well-plate wells. The next day, 100 µL of 10-fold serial dilutions of each viral sample, in media with 1% FBS, were applied to each well. Five days post infection, using the Reed and Muench method [78] viral titer was calculated by scoring the number of wells showing cytopathic effect by day.

### 4.7. Cell Viability

To determine the relative number of viable cells after treatment with Akt drugs, we used 96-well plates seeded with Vero cells as explained above. Then, we treated the cell monolayers with an increasing concentration of each compound (5 to 40 µM). At 24 and 48 hours in the presence of each compound, 20 µl of CellTiter–Blue Cell Reagent (CellTiter–Blue Cell viability assay kit) were added to the cells in each well, following the indications provided by the manufacturer (Promega, Madison, WI, USA). The cells were then incubated at 37 °C with 5% CO_2_ and the fluorescence emitted was recorded after 30 to 120 min. The number of live cells in the culture is directly proportional to the fluorescence emitted.

### 4.8. Statistical Analyses

Statistical significance was examined using GraphPad Prism 8 as described in the figure legends, and following the recommendations provided by the program. Statistical comparisons among groups were performed using the Student’s *t*-test or ANOVA tests as further detailed in each experiment. *p*-values are indicated.

## Figures and Tables

**Table 1:** Oligonucleotides used in this study.

Name	Sequence (5′->3′) ^1^	5′-end Position ^2^
pET-USUV-NS5_F	GGCGGCTAGCGGAAGACCAGGAGGAAGGAC	7684
pET-USUV-RdRp-F	GGCGGCTAGCGGGAAGCCCCAGCCACATAC	8485
pET-USUV-R	GCGGCTCGAGCAAAACCCTGTCCTCCTGGAC	10,398
pcDNA-USUV-NS5-F	AAGCTTGCCATG*TACCCATACGATGTTCCAGATTACGCT*GGAAGACCAGGAGGAAGGAC	7684
pcDNA-USUV-R	CTCGAGTTACAAAACCCTGTCCTCCTGGAC	10,398
pcDNA-USUV-RdRp-F	AAGCTTGCCATG*TACCCATACGATGTTCCAGATTACGCT*GGGAAGCCCCAGCCACATAC	8485

^1^ Sequence is in the orientation 5′ to 3′. Letters in italics represent the sequence of the HA tag. ^2^ The residue numbering corresponds to the USUV strain (939/01) (NCBI Reference Sequence NC_006551.1).

## Data Availability

The data presented in this study are available on request from the corresponding author.
